# *Staphylococcus aureus* Keratinocyte Invasion Is Dependent upon Multiple High-Affinity Fibronectin-Binding Repeats within FnBPA

**DOI:** 10.1371/journal.pone.0018899

**Published:** 2011-04-22

**Authors:** Andrew M. Edwards, Ursula Potter, Nicola A. G. Meenan, Jennifer R. Potts, Ruth C. Massey

**Affiliations:** 1 Department of Biology and Biochemistry, University of Bath, Bath, United Kingdom; 2 Microscopic Analysis Suite, University of Bath, Bath, United Kingdom; 3 Department of Biology, University of York, York, United Kingdom; 4 Department of Chemistry, University of York, York, United Kingdom; University of Liverpool, United Kingdom

## Abstract

*Staphylococcus aureus* is a commensal organism and a frequent cause of skin and soft tissue infections, which can progress to serious invasive disease. This bacterium uses its fibronectin binding proteins (FnBPs) to invade host cells and it has been hypothesised that this provides a protected niche from host antimicrobial defences, allows access to deeper tissues and provides a reservoir for persistent or recurring infections. FnBPs contain multiple tandem fibronectin-binding repeats (FnBRs) which bind fibronectin with varying affinity but it is unclear what selects for this configuration. Since both colonisation and skin infection are dependent upon the interaction of *S. aureus* with keratinocytes we hypothesised that this might select for FnBP function and thus composition of the FnBR region. Initial experiments revealed that *S. aureus* attachment to keratinocytes is rapid but does not require FnBRs. By contrast, invasion of keratinocytes was dependent upon the FnBR region and occurred via similar cellular processes to those described for endothelial cells. Despite this, keratinocyte invasion was relatively inefficient and appeared to include a lag phase, most likely due to very weak expression of α_5_β_1_ integrins. Molecular dissection of the role of the FnBR region revealed that efficient invasion of keratinocytes was dependent on the presence of at least three high-affinity (but not low-affinity) FnBRs. Over-expression of a single high-affinity or three low-affinity repeats promoted invasion but not to the same levels as *S. aureus* expressing an FnBPA variant containing three high-affinity repeats. In summary, invasion of keratinocytes by *S. aureus* requires multiple high-affinity FnBRs within FnBPA, and given the importance of the interaction between these cell types and *S. aureus* for both colonisation and infection, may have provided the selective pressure for the multiple binding repeats within FnBPA.

## Introduction

*Staphylococcus aureus* is a bacterium responsible for a wide range of superficial and invasive infections ranging in severity from mild to fatal [1]. In addition to causing severe morbidity and mortality in the healthcare environment, *S. aureus* is a growing problem in the community, causing serious infections in otherwise healthy people [2], [3]. Treatment of *S. aureus* infections is often complicated by the high prevalence of antibiotic resistant strains [4], [5]. Despite the ability of this organism to cause serious illness, *S. aureus* is primarily a commensal organism, residing within the nares and on the skin of 20–60% of the population either permanently or transiently [6]. Colonisation of the skin can lead to a number of persistent or recurring infections including, folliculitis, scalded skin syndrome, impetigo, colonisation of indwelling medical devices and wound infections [1], [7], [8].

Although originally considered an extracellular pathogen, there is both *in vitro* and *in vivo* evidence that *S. aureus* invades host cells. Although the role of invasion in colonisation and infection is unclear, it is hypothesised to facilitate evasion of immune surveillance, traversal of cellular barriers, evasion of antimicrobial therapy and to enable persistent infection [9]–[17]. Indeed, there is evidence that *S. aureus* is able to dramatically alter its phenotype (to the small colony variant phenotype) to enhance survival within host cells, which is associated with persistent infections [14], [15].

The primary mechanism by which *S. aureus* enters host cells is well characterised; staphylococcal fibronectin binding proteins (FnBPs) interact with cell surface α_5_β_1_ integrins via a fibronectin bridge [18]–[20]. It appears that FnBPA alone is sufficient for invasion since heterologous expression on the surface of otherwise non-invasive *Lactococcus lactis* or *Staphylococcus carnosus* confers the ability to invade host cells [19]. The indirect interaction of FnBPA with α_5_β_1_ integrins leads to cell signalling events, actin rearrangement and internalization of the bacterium via a mechanism that is entirely dependent on host-cell processes [11], [18], [21].

FnBPs are multifunctional proteins, comprised of distinct regions with variable binding activity. The N-terminal domain binds both fibrinogen and elastin and is implicated in biofilm formation [22], [23]. This region is followed by 11 (FnBPA) or 10 (FnBPB) non-identical fibronectin-binding repeats (FnBRs), with either high or low-affinity for fibronectin [24]. These multiple repeats enable a single FnBPA molecule to bind multiple fibronectin molecules [25]–[27]. It has been hypothesised that this facilitates bacterial interactions with multiple integrins, triggering cell signalling processes, actin rearrangement and bacterial internalisation [27]–[29]. We have previously shown that this region is essential for triggering bacterial invasion of endothelial cells [17], [30]. In addition to its role in adhesion, invasion and biofilm formation, the high prevalence of *fnb* genes amongst *S. aureus* strains suggest that FnBPs might be important for colonisation; analysis of a panel of 163 clinical isolates revealed that 22% encoded just *fnb*A, 1% just *fnb*B and 77% encoded both genes [31].

We recently investigated how the composition of the FnBR region of FnBPA affected the invasion of endothelial cells and virulence in a murine bacteremia model [17]. This study demonstrated that a single high-affinity FnBR was sufficient to trigger invasion, although this was less efficient than FnBPA variants containing multiple FnBRs [17]. Multiple FnBRs were required for virulence, enhancing bacterial dissemination into the kidneys, as well as leading to significant weight loss and death [17]. Although it is important that we understand how this protein contributes to invasive infection, this is a relatively rare event. It is more likely that the frequent interactions that occur between *S. aureus* and keratinocytes, involved in colonisation and infection of both nasal and skin surfaces [12], [13], [32], are responsible for selection of FnBPA function. We therefore investigated the role of the FnBR-region in the adhesion to, and invasion of, keratinocytes.

## Methods

### Bacterial strains and growth conditions

A detailed list of the strains used in this study is presented in Table 1. *S. aureus* 8325.4 strains were cultured in Tryptic Soy Broth (TSB, Oxoid) at 37°C in air with shaking for 16 h. *S. aureus* CFU were quantified on Tryptic Soy Agar (TSA, Oxoid) plates incubated overnight at 37°C in air. *L. lactis* strains were cultured in M17 broth (supplemented with 0.5% w/v glucose) for 16 h at 30°C in air (with the indicated concentration of nisin). As required, bacteria were incubated in the presence of the following antibiotics: Chloramphenicol 10 µg ml^−1^ (*S. aureus*) or Erythomycin 5 µg ml^−1^ (*L. lactis*) or 250 µg ml^−1^ (*E. coli*).

**Table 1:** Strains and plasmids used in this study.

Species/strain/plasmid	Relevant characteristics	Source/Reference
***E. coli***		
(pMSP7517)	Nisin-inducible vector containing the *prg*B gene of *Enterococcus faecalis*	[51]
K12 ER2925	Cloning host	New England Biolabs
BL21	Recombinant peptide expression host	
pGEX-6P-2	Expresses GST-tagged FnBR9,10 peptide	This study
***Staphylococcus aureus***		
DU5883	*fnbA- fnbp-* isogenic mutant of 8325.4	[52]
DU5883 (pFnBA4)		
(referred to here as pFnBPR1–11)	*fnbp* - strain complemented with the plasmid pFnBA4 expressing full-length FnBPA: FnBPR1–11	
pFnBPR0	Expresses FnBPA variant containing no Fn-binding repeats: FnBPR0	[17]
pFnBPR1	Expresses FnBPA variant containing Fn-binding repeat 1 only: FnBPR1	
pFnBPR11	Expresses FnBPA variant containing Fn-binding repeat 11 only: FnBPR11	
pFnBPR10,11	Expresses FnBPA variant containing Fn-binding repeats 10 & 11: FnBPR10& 11	
pFnBPR1,10,11	Expresses FnBPA variant containing Fn-binding repeats 1, 10 & 11: FnBPR1, 10, 11	
pFnBPR9–11	Expresses FnBPA variant containing Fn-binding repeats 9–11: FnBPR9–11	
pFnBPR2	Expresses FnBPA variant containing Fn-binding repeat 2 only: FnBPR2	
pFnBPR8	Expresses FnBPA variant containing Fn-binding repeat 8 only: FnBPR8	
pFnBPR7,8	Expresses FnBPA variant containing Fn-binding repeats 7 & 8: FnBPR7,8	
pFnBPR6–8	Expresses FnBPA variant containing Fn-binding repeats 6–8: FnBPR6–8	
***Lactococcus lactis***		
*L. lactis* NZ9800	*nis*A defective isogenic mutant of NZ9700 (*fnb*-)	[53]
pMFnBPR1–11	Nisin controlled expression of FnBPA containing repeats 1–11: FnBPR1–11	[17]
pMFnBPR1	Nisin controlled expression of FnBPA containing repeat 1: FnBPR1	
pMFnBPR1,10,11	Nisin controlled expression of FnBPA containing repeats 1,10,11: FnBPR1,10,11	
pMFnBPR6–8	Nisin controlled expression of FnBPA containing repeats 6–8: FnBPR6–8	This study

*S. aureus* 8325.4-derived Δ*fnb*A/B expressing *fnb*A variants from a plasmid have been characterised previously [17] to establish equal surface expression levels between strains expressing each construct. All constructs have identical N-terminal and C-terminal domains, varying only in the composition of the FnBR-region.

### Recombinant FnBR expression

A polypeptide corresponding to high-affinity repeats FnBR9,10 (FnBPA residues 763–838; Swiss-Prot entry P14738) was expressed and purified in a manner similar to that previously described for single FnBRs [24]. The GST-tag was removed using 3C protease and FnBR9,10 was further purified by reversed-phase HPLC.

### Construction and controlled expression of FnBPA variants in *L. lactis*

Expression levels of FnBPA variants in *S. aureus* and *L. lactis* have been determined previously [17]. With the exception of the pMR6-8 construct, all nisin-inducible FnBPA constructs have been described previously [17]. The pMR6-8 construct was made as described previously using standard molecular biology techniques [17], [33]. Briefly, the enitre R6-8 *fnb*A variant was amplified from pFnBPR6-8 using primers containing NcoI and XhoI sites (AAACCATGGAGGAGGTATTATAGTGAAAAACAATCTTAGG) (AAACTCGAGCTAACTTTATCTCTCAGTTCGTTATC) and ligated into similarly digested pMSP7517 plasmid. Ligated constructs were transformed into CaCl_2_ treated *E. coli* K12 ER2925. Plasmids were recovered from the *E. coli* transformants (the DNA sequence was confirmed) and transformed into *L. lactis* NZ9800. As all constructs contained the A domain (Table 1), expression was confirmed by assessment of fibrinogen binding [17]. Previous work has determined that nisin controlled expression of FnBPA variants in *L. lactis* using this system produces equal expression levels between strains at identical nisin concentrations [17].

### Cell culture

The spontaneously immortalised keratinocyte cell line HaCat was used because it closely resembles primary keratinocyte cells and has been used previously in studies of *S. aureus*-keratinocyte interactions [34]–[37]. All cells were cultured in Dulbecco's modified Eagle's medium (Invitrogen), with calcium adjusted to 2.8 mM and supplemented with FBS (10%) and l-glutamine (2 mM) at 37°C and 5% CO_2_, conditions that support differentiation, keratinization and tight-junction formation [38].

Cells were cultured in T75 flasks to approximately 95% confluency, liberated with trypsin-EDTA, resuspended in culture medium and added to 24-well plates containing thermanox glass coverslips [30]. Plates were incubated for 48 h as described above before the coverslips were removed, dip washed in (phosphate buffered saline)PBS and added to new 24-well plates containing fresh medium and bacteria [30]. For experiments using inhibitors of cell function, these were incubated with the cells 60 min prior to the addition of bacteria and concentrations maintained during the assay; Genistein (200 µM), Wortmannin (20 nM), cytochalasin D (50 µM), PP2 (10 µM), cholchicine (1 µM), cycloheximide (25 µM) and methyl-β-cyclodextrin (2 mM) [17]. EA.Hy926 cells were cultured and prepared as previously described [17].

### Cell attachment and invasion assays

Attachment and invasion assays were performed as described before [17]. Cultured cells were dissociated from plastic flasks using trypsin-EDTA solution (Invitrogen) and approximately 5×10^5^ (in 0.5 ml medium) were seeded into each well of 24-well plates (Nunc) containing 13 mm plastic Thermanox™ cover slips (Fisher) and allowed to attach for 48 h (37°C, 5% CO_2_). Coverslips were dip-washed once in PBS and placed in the well of a new 24-well plate containing 490 µl of DMEM containing 10% FBS. To each well, 10 µl of washed bacteria were added (approximately 1×10^7^ CFU *S. aureus* and 5×10^7^ *L. lactis*) and incubated for 15–90 minutes at 37°C in 5% CO_2_.

To measure the total number of bacteria associated with the cells (adherent and internalized – referred to in the text and figures as adhesion), coverslips were dip-washed three times in PBS and added to wells containing 500 µl 0.5% Triton X-100. Wells containing coverslips were agitated by pippetting to fully lyse the cells and CFU were enumerated by serial dilution and plating onto TSA agar plates and incubation overnight at 37°C.

For invasion assays, the bacterial suspension was removed and replaced with 500 µl DMEM/10% FBS supplemented with 200 µg ml^−1^ gentamicin and incubated at 37°C in 5% CO_2_ for 60 min. Coverslips were washed three times in PBS, lysed and plated for CFU as described for the adhesion assay above. In assays where metabolic inhibitors were used, these were added to cell monolayers for 60 min prior to the experiment and concentrations maintained during incubation with bacteria.

### Determination of α_5_β_1_ integrin expression by keratinocytes and endothelial cells

HaCat Keratincoytes and EA. Hy926 endothelial cells were cultured in T75 flasks to approximately 95% confluency, as described above. Cells were liberated from the flasks by the use of non-enzymatic cell dissociation solution (4 ml, Sigma) and collected by centrifugation (2,000× *g*, 10 min). Cells were subsequently resuspended in lysis buffer (1% TX-100, 100 units ml^−1^ DNase, protease inhibitor cocktail (Sigma) in PBS) and incubated for 20 min at 37°C. Insoluble debris was pelleted by centrifugation and the supernatant recovered. Total protein was determined using the Bradford assay and 20 µl aliquots containing 10 µg protein mixed with Laemmli sample buffer [33] before boiling for 5 min. Samples were then subjected to SDS-PAGE on duplicate 10% acrylamide gels. Total protein was detected by the use of EZ-blue protein detection reagent (Sigma). Alternatively, proteins were transfered to nitrocellulose membrane using a Bio-Rad semi-dry blotter. The membrane was subsequently blocked in PBS containing 10% skimmed milk powder for 1 hr at room temperature. The protein bands corresponding to α_5_ or β_1_ integrin subunits were detected using rabbit polyclonal anti-integrin α_5_ (H-104) or β_1_ (M-106, both from Santa Cruz Biotechnology) antibodies and then HRP-conjugated protein A (Sigma). Bound antibody/protein A was detected using enhanced chemiluminescence reagent (GE Healthcare).

### Scanning electron microscopy

*S. aureus* was incubated with keratinocytes or endothelial cells as above for invasion assays for 15–90 minutes. Coverslips were then washed twice with serum-free DMEM before fixation with DMEM containing 2.5% gluteraldehyde and 10 mg ml^−1^ potassium ferrocynaide for 2 hr at 37°C in 5% CO_2_. Cells were postfixed in a solution of 1% osmium tetroxide and 1% potassium ferrocyanide for 1 hr in air at room temperature. Coverslips were washed twice in distilled water over 10 mins before staining with 2% uranyl acetate for 1 hr in the dark. Cells were subsequently dehydrated in increasing concentrations of acetone (50–100%) followed by 1∶1 acetone: hexamethyldisilazane and finally 100% hexamethyldisilazane, which was allowed to evaporate over 1–2 hrs in air. Samples were then examined using a JEOL JSM6480LV scanning electron microscope.

### Statistics

For adhesion and invasion assays, statistical analyses were performed with Student's *t* test using the Bonferroni correction for multiple comparisons [17]. Values that were statistically significantly different from controls are indicated by asterisks in the figures. Error bars indicate the mean average ± standard deviation of multiple independent experiments (indicated in the figure legend).

## Results

### *S. aureus* 8325.4 adhesion to keratinocytes is similar to endothelial cells but invasion levels are significantly lower

**Figure 1:**
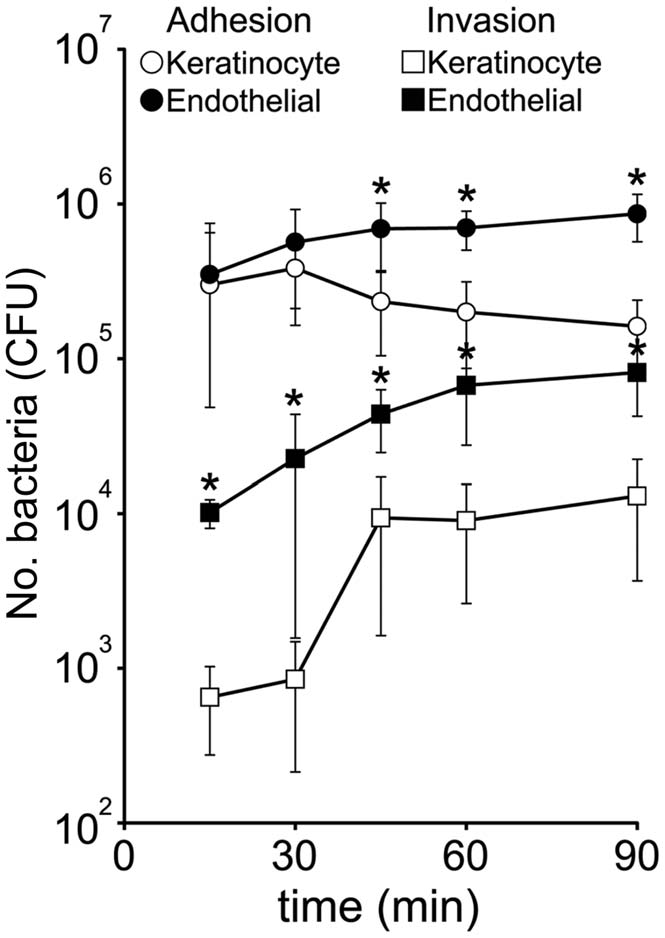
*S. aureus* adhesion to, and invasion of, keratinocyte and endothelial cells over time. The total number of *S. aureus* 8325.4 (DU5883 Δ*fnb*A/B pFnBA4 expressing full length FnBPA) associated (adherent and internalized; circles) and internalized (squares) with each cell type was determined over a period of 90 minutes. Values for adhesion to, and invasion of, endothelial cells that are statistically significantly different from those obtained with HaCat cells are denoted (*). Experiments were performed 3 times in duplicate. Multiplicity of infection (MOI) = 20. Error bars represent the standard deviation of the mean.

*S. aureus* attachment to the skin is the precursor to colonisation and infection. Invasion is also likely to play an important role, particularly in maintaining persistent or recurring infection [13]. We employed the well-characterised keratinocyte cell line HaCat as a model to study the role of FnBPA in bacterial adhesion to, and invasion of, the skin and nares. To determine the kinetics of keratinocyte-*S. aureus* interactions, we measured adhesion to and invasion of HaCat keratinocytes over time, until saturation levels were observed (Fig. 1). Adhesion occurred rapidly, with approximately 3×10^5^ CFU (∼3% inoculum) *S. aureus* attached after 15 minutes (Fig. 1). This number did not change significantly over time (up to 90 mins), suggesting that all available binding sites on the host cells were occupied (Fig. 1). Invasion, by contrast, occurred more slowly. After 15 mins <10^3^ CFU *S. aureus* had internalised, despite the high number of adherent bacteria and there was no significant increase up to 30 mins, indicating that the invasion process includes a lag-phase (Fig. 1). However, between 30 and 45 mins this number increased >10-fold to ∼10^4^ CFU (∼0.1% inoculum). There was no further increase in the number of internalised *S. aureus* up to 90 minutes, indicating that maximal invasion levels had been reached (Fig. 1).

To compare how invasion of keratinocytes compared to endothelial cells we also examined the adhesion to and invasion of EA. hy926 endothelial cells over the same time period (Fig. 1). *S. aureus* adhesion to endothelial cells was identical to that of keratinocytes after 15 minutes (Fig. 1). Adhesion to the endothelial cells increased over time and was significantly greater than HaCat cells after 45 mins. *S. aureus* invasion of endothelial cells was, however, 16-fold greater than that of keratinocytes after 15 minutes (Fig. 1). Endothelial cell invasion continued to increase until reaching saturation at 45–60 mins, at which point it was still approximately 6-fold greater than that seen with keratinocytes.

### Low level keratinocyte invasion is not due to different invasion processes being utilised by *S. aureus*

**Figure 2:**
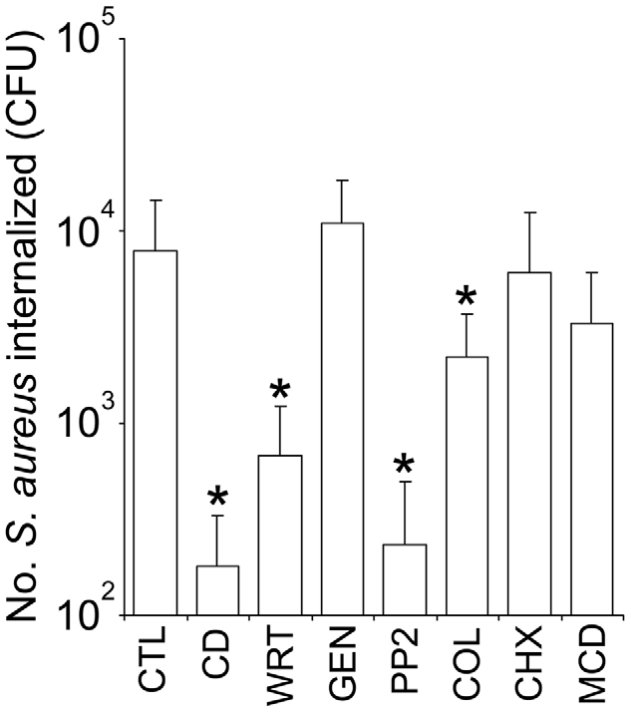
Keratinocyte invasion occurs via a similar mechanism to endothelial cells. HaCat cells were pre-incubated (60 min) with inhibitors of cell function before the addition of *S. aureus* FnBPAR1–11 (DU5883 Δ*fnb*A/B pFnBA4) and invasion determined after 90 mins. Inhibitors used were cytochalasin D (CD, inhibits actin polymerization), wortmannin (WRT, inhibits PI3-Kinase activity), genistein (GEN, inhibits tyrosine kinase activity), PP2 (PP2, Src kinase inhibitor), colchicine (COL, interferes with microtubule organisation), cycloheximide (CHX, inhibits eukaryotic protein synthesis) and methyl-β-cyclodextrine (MCD, depletes membrane cholesterol). Inhibitor-free medium was used as a positive control (CTL). Experiments were performed three times in duplicate. MOI = 20. Error bars represent the standard deviation of the mean. Values that are significantly different (p = <0.05) from control are indicated (*).

Although several different pathogenic bacteria utilize integrins to trigger invasion, the cellular mechanisms involved can vary significantly. For example, *Streptococcus pyogenes* can invade via both caveolae and membrane ruffling depending on the invasin [39], [40]. To examine whether the difference in efficiency of invasion of keratinocytes when compared to endothelial cells is due to the bacteria utilizing a different cellular process, we measured the internalisation of *S. aureus* FnBPR1–11 by HaCat cells pre-treated with inhibitors of cell function used previously in studies with endothelial cells [17]. Disruption of host cell actin rearrangement (cytochalasin D), PI3K signalling (wortmannin), Src kinase signalling (PP2) or microtubule function (colchicine) all significantly inhibited *S. aureus* invasion, albeit to differing degrees (Fig. 2). By contrast, inhibition of tyrosine kinases (genistein), de novo protein synthesis (cycloheximide) or depletion of cholesterol (methyl-β-cyclodextrin) did not significantly affect invasion (Fig. 2). This was identical to that found when endothelial cells were studied [17], demonstrating that differences in these cellular process were not responsible for the difference in cell invasion efficiency observed.

**Figure 3:**
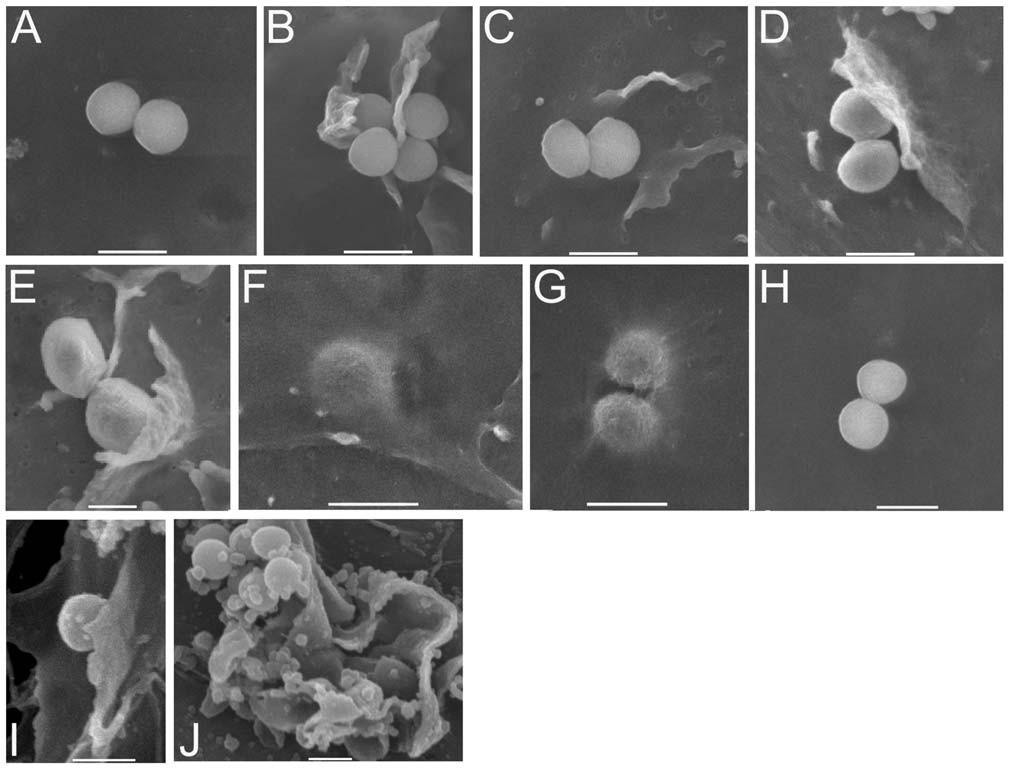
*S. aureus* FnBPR1–11 induces membrane ruffling and invasion of keratinocytes. *S. aureus* FnBPR1–11 (DU5883 Δ*fnb*A/B pFnBA4) was incubated with keratinocytes for 15 (A), 30 (B–E) or 90 mins (F,G) before washing, fixation, processing and examination by scanning electron microscopy. As a negative control, *S. aureus* FnBPR0 was also examined after 90 mins (H). As a comparison, the interaction of *S. aureus* FnBPR1–11 with EA. Hy926 endothelial cells after 30 mins was also visualised (I–J). In each case the bar represents 1 µm. MOI = 20.

To confirm the role of cellular processes in invasion, we examined *S. aureus*-keratinocyte interactions using scanning electron microscopy (Fig. 3). After 15 minutes' incubation with *S. aureus* FnBPR1–11, there were significant numbers of attached bacteria but there were no visible changes in the host cell surface (Fig. 3A). After 30 minutes, a few adherent bacteria were associated with host-cell membrane alterations consistent with actin rearrangement (Fig. 3B–E). These morphological changes in the host cell membrane were consistent and we did not observe other types of changes, such as the formation of caveolae.

By 90 minutes post inoculation, a number of fully internalised bacteria could be observed within the keratinocytes (Fig. 3F,G). By contrast to *S. aureus* FnBPR1–11, *S. aureus* FnBPR0 did not induce any membrane alterations after 90 mins (Fig. 3H). We also examined the interaction of *S. aureus* FnBPR1–11 with EA. Hy926 cells. After 30 mins adherent bacteria were associated with membrane ruffles, similar to those observed with keratinocytes (Fig. 3I,J).

### The composition of the FnBR region significantly affects invasion of keratinocytes

**Figure 4:**
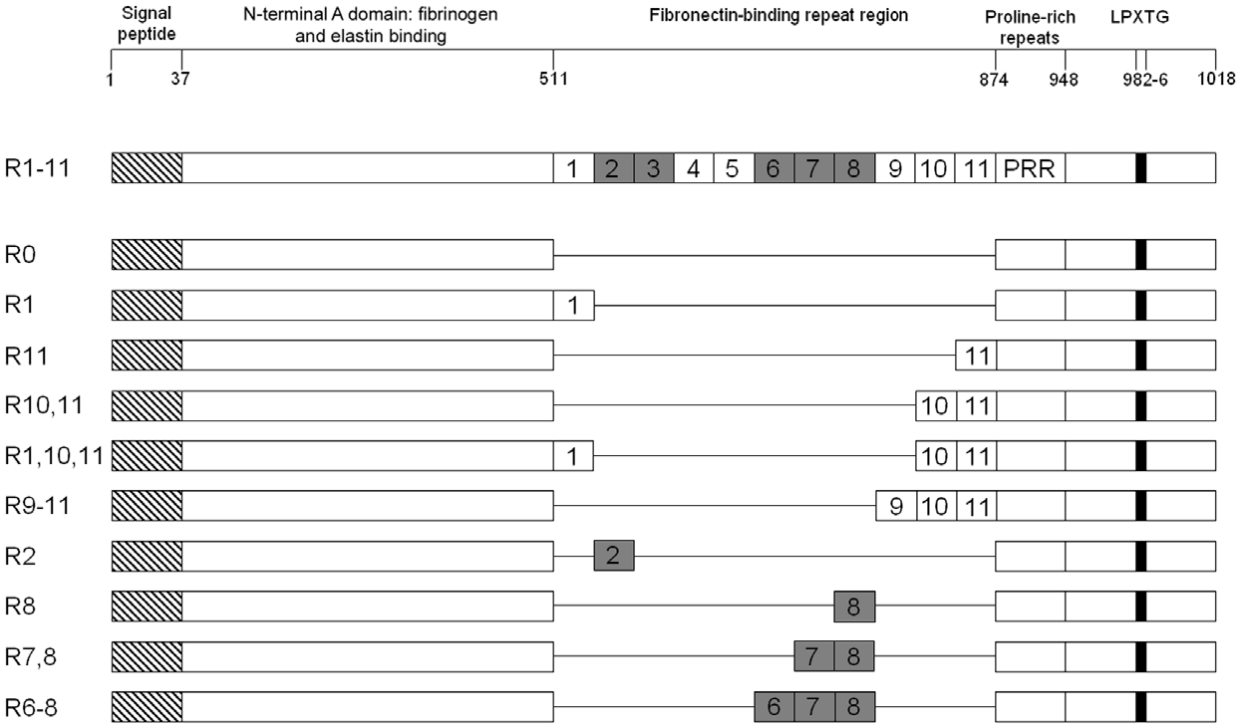
Diagrammatic representations of FnBPA and the variant constructs used in this study. The relative positions of each of the major functional domains of FnBPA from *S. aureus* 8325.4 (SWISS-Prot P14738) (not to scale) are indicated, along with a schematic of each of the FnBPA variant constructs used in this study, which vary only in the composition of the FnBR domain. Low-affinity FnBRs are shaded.

**Figure 5:**
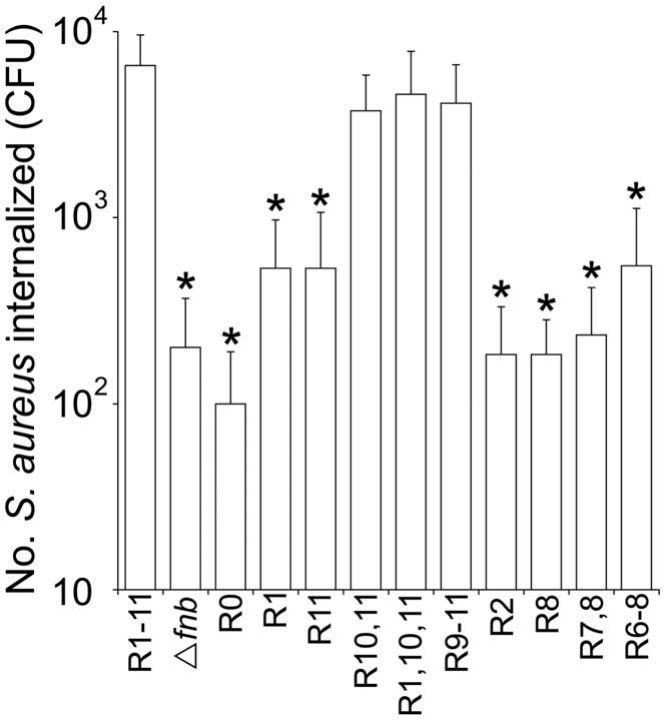
The composition of the FnBR domain of FnBPA modulates host cell invasion. Keratinocytes were incubated with either *S. aureus* DU5883 Δ*fnb*A/B (Δ*fnb*) or *S. aureus* Δ*fnb* expressing one of the FnBPA variant constructs detailed in figure 4 for 90 minutes and the number of internalized bacteria determined. Experiments were performed 3 times in duplicate. Error bars represent the standard deviation of the mean. MOI = 20. Values that are significantly different (p = <0.05) from *S. aureus* Δ*fnb* are indicated (*).

In a previous study we showed that a single high-affinity Fn binding repeat (FnBR) in FnBPA was sufficient for invasion of endothelial cells [17]. To assess the role of FnBPA, and in particular the FnBR region, we examined adhesion to and invasion of HaCat cells by *S. aureus* expressing full length FnBPA: FnBPR1–11, a Δ*fnb*A/B mutant and *S. aureus* pFnBPR0 (which lacks the FnBR region) after 90 minutes (Fig. 4). Adhesion of *S. aureus* to HaCat cells was unaffected by the presence or absence of FnBPA (data not shown). Invasion, however, was dependent on the presence of FnBPA; *S. aureus* lacking FnBPs invaded at levels 15 times lower than *S. aureus* FnBPR1–11 (Fig. 5). FnBPA-triggered invasion was entirely dependent of the presence of the FnBR region; *S. aureus* FnBPR0 invaded HaCat cells at levels identical to *S. aureus* Δ*fnb*A/B (Fig. 5).

To examine how the composition of the FnBR region modulates invasion, we measured internalisation of *S. aureus* expressing FnBPA variants containing various numbers of high- or low-affinity tandem repeats (Fig. 4). Previous work has demonstrated that these variants are expressed on the surface of *S. aureus* at equal levels and characterised the fibronectin-binding of each construct [17]. Although *S. aureus* FnBPR1 or FnBPR11 invaded at levels significantly higher than *S. aureus* FnBPR0 this was >12-fold less than *S. aureus* FnBPR1–11 (Fig. 5). The presence of a second high affinity repeat (FnBPR10,11) was required to enhance internalisation to levels that were similar to that of *S. aureus* FnBPR1–11 (Fig. 5). Three high-affinity repeats (FnBPR1,10,11 and FnBPR9–11) also triggered invasion at similar levels to FnBPR1–11 (Fig. 5).

In contrast to the FnBPA variants with high-affinity repeats, *S. aureus* expressing FnBPA variants containing a single (FnBPR2 or FnBPR8) or two (FnBPR7,8) low-affinity repeats did not invade at levels that were significantly greater than FnBPR0 (Fig. 5). *S. aureus* expressing a FnBPA variant with three tandem low-affinity repeats (FnBPR6–8) did invade at levels above that of FnBPR0 but these were approximately 12-fold lower than *S. aureus* FnBPR1–11 (Fig. 5).

### Over-expression of FnBPR1 or FnBPR6–8 fails to confer maximal invasion

**Figure 6:**
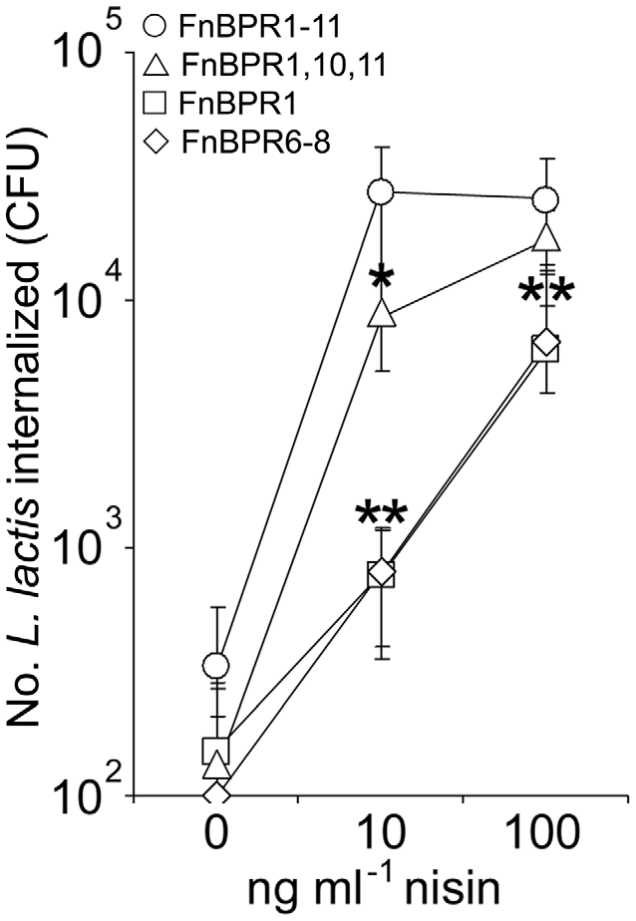
Over-expression of FnBPR1 or FnBPR6–8 fails to trigger maximal invasion of Keratinocytes. Expression of FnBPA variant constructs on the surface of *L. lactis* was performed using a nisin-inducible system. Invasion of *L. lactis* expressing FnBPAR1–11 (circles), FnBPAR1,10,11 (triangles), FnBPAR1 (squares) or FnBPAR6–8 (diamonds) was determined at 3 different levels of induction (0, 10 or 100 ng ml^−1^ nisin). Experiments were performed 3 times in duplicate. Error bars represent the standard deviation of the mean. MOI = 100. Values that are significantly different (p = <0.05) from *L. lactis* FnBPAR1–11 at identical nisin concentrations are indicated (*).

We have shown previously that FnBPA-mediated invasion of endothelial cells is dependent on the total number of FnBRs on the bacterial cell surface, rather than the number within an individual FnBPA molecule [17]. As such, high-level expression of FnBPR1 can trigger endothelial cell invasion to similar levels as *S. aureus* expressing FnBPR1–11. To determine whether maximal invasion of keratinocytes (FnBPR1–11 levels) could be triggered by over-expression of otherwise weakly-invasive FnBPR1 or FnBPR6–8 constructs, or whether invasion is absolutely dependent on multiple FnBRs within FnBPA, we employed a nisin-inducible system to control and over-express FnBPA variants on the surface of *L. lactis* [17]. Previous work [17] has demonstrated that, at identical concentrations of nisin, expression levels of FnBPA variants on the surface of *L. lactis* are equal. *L. lactis* expressing each FnBPA construct at three surface expression levels (by inducing expression at three concentrations of nisin; 0, 10 or 100 ng ml^−1^) were assessed for invasion of HaCat cells (Fig. 6). At the lowest level of expression (0 ng ml^−1^ nisin, at which low-level expression occurs due to a leaky promoter) there were only negligible levels of invasion by *L. lactis* expressing each of the FnBPA variants (Fig. 6). Induction of FnBPR1–11 expression (10 ng ml^−1^ nisin) increased invasion 80-fold to levels similar to those seen with *S. aureus* FnBPR1–11 (Figures 1 and 5). Greater induction of FnBPR1–11 expression (100 ng ml^−1^) did not increase internalisation suggesting that maximal invasion levels had been reached (Fig. 6). Invasion of *L. lactis* FnBPR1,10,11 also increased dramatically when induced at 10 ng ml^−1^ nisin (relative to no induction), but at significantly lower levels than *L. lactis* FnBPR1–11 (Fig. 6). However, at the highest level of expression, *L. lactis* R1,10,11 was internalised at the same level as FnBPR1–11 (Fig. 6), mimicking the data obtained with *S. aureus* expressing FnBPA variants (Fig. 5). Induction of FnBPR1 or FnBPR6–8 expression (10 ng ml^−1^ nisin) promoted *L. lactis* invasion but at levels >30-fold lower than *L. lactis* FnBPR1–11 (Fig. 6). Further induction of FnBPR1 or FnBPR6–8 expression (100 ng ml^−1^ nisin) further increased invasion, but this was still 4–fold lower than that seen for *L. lactis* FnBPR1–11 at the same level of induction (Fig. 6).

### Inefficient invasion of keratinocytes may be due to weak α_5_β_1_ integrin expression

**Figure 7:**
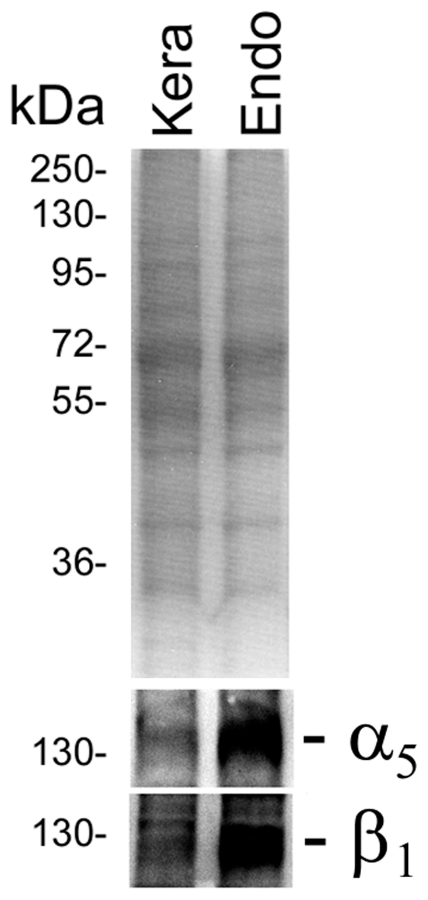
Keratinocyte expression of α_5_ and β_1_ integrins is lower than endothelial cells. HaCat keratinocytes (Kera) or EA. Hy926 endothelial (Endo) cells were harvested from flasks, lysed and the lysate examined by SDS-PAGE and Western-immunoblot to determine relative α_5_ or β_1_ integrin expression levels.

Although invasion processes do not seem to vary between endothelial cells and keratinocytes, a higher number of high affinity FnBRs are required for efficient invasion of keratinocytes. As such we hypothesised that the difference between the invasion efficiency of keratinocytes and endothelial cells may be due to differences in the density of the host cell ligand, the cell surface α_5_β_1_ integrin. To test this, we compared the expression of the α_5_ and β_1_ integrin subunits in keratinocytes and endothelial cells by performing a Western-immunoblot of whole-cell protein extracts. This revealed that α_5_β_1_ integrin expression levels were significantly higher in endothelial cells than keratinocytes (Fig. 7). This is consistent with studies of human skin [41], and may explain the differences in invasion levels between the two cells types.

### A FnBR peptide inhibits *S. aureus* invasion of keratinocytes

**Figure 8:**
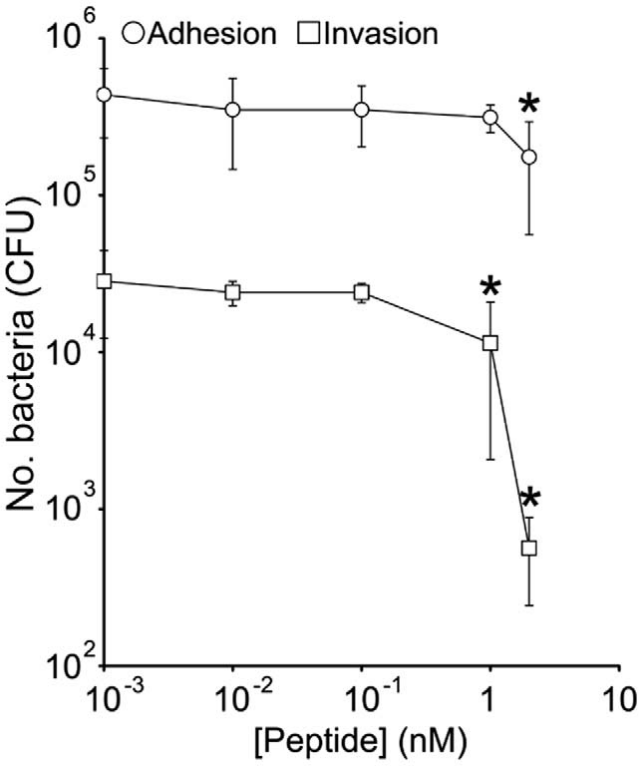
A recombinantly-expressed FnBR peptide inhibits *S. aureus* invasion. The adhesion to (circles), and invasion (squares) of, keratinocytes by *S. aureus* FnBPR1–11 (DU5883 Δ*fnb*A/B pFnBA4) in the presence of various concentrations of recombinant FnBPA peptide (R9,10). Values that statistically significantly difference from those obtained in the absence of peptide are indicated (*). MOI = 20. Experiments were performed 3 times in duplicate. Error bars represent the standard deviation of the mean.

The rise in antibiotic resistance has prompted the search for novel approaches to preventing bacterial colonisation and infection. As there seems to be a critical ratio of FnBR and host ligand needed to invade cells ([17] Fig. 5 and 6), we hypothesized that an FnBR peptide might be sufficient to outcompete FnBPA and prevent *S. aureus* invasion. A tagless recombinantly-expressed peptide corresponding to FnBR9,10 (two high-affinity repeats) was used in an assay to examine its potential to block adhesion and invasion (Fig. 8). The peptide reduced adhesion approximately two-fold at the very highest concentration, despite FnBPA not being required for attachment of keratinocytes (Fig. 8, data not shown). By contrast, the peptide significantly inhibited *S. aureus* invasion at concentration of 1 nM (>2-fold reduction), which was even more pronounced at 2 nM (>5-fold) (Fig. 8). As such, this peptide may warrant further study as a potential novel prophylactic agent.

## Discussion

*S. aureus* is able to colonise and infect skin, causing a wide spectrum of infections ranging from superficial to invasive, acute to chronic [1]. Infection can occur in healthy individuals as well as those with broken skin due to wounds, inserted medical devices or chronic skin conditions such as psoriasis [8]. Central to colonisation and infection is the interaction of *S. aureus* with keratinocytes, which form an important barrier between the internal organs and external environment. Adhesion of *S. aureus* to keratinocytes results in inflammatory cytokine release and stimulates secretion of several antimicrobial peptides, of which β-defensin 3 reaches levels sufficient to kill *S. aureus* [42], [43]. Cellular invasion is apparently not necessary to trigger these responses but internalised bacteria cause necrotic and apoptotic cell death [37]. Interestingly, *S. aureus* adhesion to endothelial cells was equal to that of keratinocytes after 15 minutes, but was significantly greater after 90 minutes. This may reflect a decrease in keratinocyte-attached CFU due to the release of antimicrobial peptides by the HaCat cells.

Although the role of cellular invasion in colonisation and pathogenesis is unclear, previous work has shown that *S. aureus* can persist within keratinocytes for extended periods and it is possible that invasion provides shelter from the host antimicrobial arsenal. Intracellular *S. aureus* has been identified in chronic infections where it could form a protected reservoir as well as a mechanism of cellular dissemination and penetration of deeper tissues [14], [15].

We have shown previously that endothelial cell invasion is dependent upon the surface density of the FnBRs within FnBPA. The presence of multiple repeats within FnBPA is equivalent to multiple invasins in a single molecule, significantly enhancing efficiency and reducing immune exposure [17].

As the interaction of *S. aureus* with keratinocytes is a considerably more common event than with endothelial cells, we hypothesized that it likely provides the main selective pressure for the composition of FnBPs. The lack of a role for FnBPA in adhesion to HaCat keratinocyte cells is not surprising since *S. aureus* encodes a large number of adhesins. Indeed, at least five different *S. aureus* surface proteins are implicated in attachment to nasal epithelial cells, including ClfB, IsdA, SdrC, SdrD and SasG, as well as wall teichoic acid, which is essential for nasal colonisation [44]–[47]. By contrast, invasion of HaCat keratinocytes was FnBPA-dependent and, in keeping with previous work [17], the FnBR region was essential. Previous work has shown that a single high-affinity, or three low-affinity repeats were sufficient to trigger maximal invasion of endothelial cells [17]. By contrast, our data indicate that keratinocyte invasion requires much higher surface densities of FnBRs than for invasion of endothelial cells. This may be a result of the significantly lower level of α_5_β_1_ integrin expression by the keratinocyte cells, which is in keeping with *in vivo* analysis of normal human skin [41]. It is therefore possible that the difference in the speed of invasion is due to the increased length of time required for *S. aureus* to engage with sufficient α_5_β_1_ integrins on the keratinocyte cell surface and for subsequent cell-signalling events to occur. Additionally, the presence of multiple FnBRs within FnBPA may increase the efficiency of Fn binding through cooperative binding to arrays of FnBRs as observed recently for a Fn-binding protein from *Streptococcus pyogenes* [48]. It is also possible that delayed entry of *S. aureus* into keratinocytes is desirable for the bacterium. A previous report [49] indicated that *S. aureus* delays uptake into endothelial cells in order to have sufficient time to prepare for intracellular life via up-regulation of e.g. toxin genes. It is possible that *S. aureus* employs a similar strategy for keratinocytes.

Although *S. aureus* invaded the endothelial cells more readily than the keratinocytes, the mechanism used appears to be the same (Figure 2 and [17]). Using identical concentrations of cell-function inhibitors used previously to study endothelial cell invasion [17], entry of *S. aureus* into HaCat cells was also found to involve PI3 and Src-kinases as well as actin rearrangement. Whilst one might expect genistein to have a similar effect to PP2, our data are not the first to suggest that genistein does not inhibit all the targets of PP2. Indeed, this phenomenon has been observed previously in studies of invasion mediated by fibronectin-binding protein invasins of *S. pyogenes*. Wang et al. [50] showed that invasion of epithelial cells by *L. lactis* expressing M-protein could be inhibited by PP2 but not by genistein. Conversely, invasion by *L. lactis* expressing the invasin SfbI was inhibited by genistein but not PP2. Interestingly, SfbI-mediated invasion relies on caveolae whilst M-protein mediated invasion does not, which ties in with our data showing no inhibition by methyl-β-cyclodextrin. It appears that, using our model system, FnBPA-mediated invasion is highly similar to that of *S. pyogenes* M-protein, rather than the structurally similar SfbI.

In summary, keratinocytes are significantly less amenable to invasion by *S. aureus* than endothelial cells, and require multiple repeats within FnBPA for invasion. As such, *S. aureus* interactions with keratinocytes and similar cells may provide the selection pressure for the multiple FnBRs within FnBPs. The poor penetration of many antibiotics into cells means that intracellular *S. aureus* could represent a reservoir for persistent infection [13], [14]. Our data, in keeping with previous reports [18], [30], strongly suggest that FnBR peptides are highly effective at reducing *S. aureus* invasion and might form a novel prophylactic approach to reducing carriage and/or the development of chronic infections. Such an approach may have the added benefit of preventing invasion by other skin colonising pathogens such as *S. pyogenes*, which employ similar mechanisms of internalisation [11].
